# METABOLOME-WIDE STUDY OF CVD IN SWEDISH TWINS

**DOI:** 10.1093/geroni/igae098.2296

**Published:** 2024-12-31

**Authors:** Asmus Cosmos Skovgaard, Jonathan K L Mak, Sara Hägg, Juulia Jylhävä, Mette Soerensen

**Affiliations:** University of Southern Denmark, Odense M, Syddanmark, Denmark; Karolinska Institutet, Stockholm, Stockholms Lan, Sweden; Karolinska Institutet, Stockholm, Stockholms Lan, Sweden; University of Tampere, Tampere, Pirkanmaa, Finland; University of Southern Denmark, Odense M, Syddanmark, Denmark

## Abstract

Globally, cardiovascular diseases (CVD) are the leading cause of death, with coronary artery disease (CAD) and stroke as the most frequent diseases. The molecular basis of CVD is potentially confounded by genetic and environmental factors, an issue which is reduced by investigating intra-pair differences in twins. Here, we study 11,228 twins with nuclear magnetic resonance-based metabolome data and register based diagnoses on incident CAD and stroke collected from the Swedish Patient Register. Statistical analyses were performed both at the individual level and twin-pair level by Cox regression analysis. Biomarkers being significant (FDR< 0.05) both at the individual level and twin pair level in analysis of CAD were identified and LASSO analysis was used to identify the most influential independent biomarkers. A more explorative approach was taken for stroke by applying a cut off of p value< 0.05. Different biomarkers were found for CAD and stroke, which can indicate that the biological mechanisms are partly distinct in the two diseases. We found that ApoB-containing lipoproteins are more positively associated with CAD, which might be due to their pro-atherogenic effects. Contrary, ApoA1-containing lipoproteins are more negatively related to stroke possibly due to their anti-atherogenic effects and potential ability to cross the blood-brain-barrier, an ability not shared with the ApoB-containing lipoproteins. In conclusion, we present novel biomarkers for CAD and stroke by performing metabolomic analyses in twins, thereby lowering the confounding induced by shared genetic and environmental factors, a study design which is rare within the CVD field.

